# Association between *CORIN* promoter methylation and stroke: Results from two independent samples of Chinese adults

**DOI:** 10.3389/fneur.2023.1103374

**Published:** 2023-03-31

**Authors:** Linan Chen, Jun Jiang, Jialing Yao, Ying Lu, Xiaolong Zhang, Mingzhi Zhang, Qiu Zhang, Hao Peng

**Affiliations:** ^1^Department of Epidemiology, School of Public Health, Medical College of Soochow University, Suzhou, China; ^2^Department of Tuberculosis Control, Suzhou Center for Disease Control and Prevention, Suzhou, China; ^3^Department of Chronic Disease, Gusu Center for Disease Control and Prevention, Suzhou, China; ^4^Jiangsu Key Laboratory of Preventive and Translational Medicine for Geriatric Diseases, Suzhou, China

**Keywords:** Corin, DNA methylation, stroke, prospective observational study, independent replication

## Abstract

**Objective:**

As the physical activator of natriuretic peptides, corin has been associated with stroke, but the underlying mechanism is not very clear. Here, we examined whether the *CORIN* promoter’s methylation, an epigenetic DNA modification, was associated with the risk of stroke in two independent samples.

**Methods:**

A total of 1771 participants including 853 stroke cases and 918 healthy controls were included as a discovery sample and 2,498 community members with 10 years of follow-up were included as a replication sample. DNA methylation of the *CORIN* promoter was quantified by target bisulfite sequencing in both samples. We first examined the single CpG association, followed by a gene-based analysis of the joint association between multiple CpG methylation and stroke, adjusting for conventional risk factors.

**Results:**

The single CpG association analysis found that hypermethylation at all of the 9 CpG sites assayed was significantly associated with lower odds of prevalent stroke in the discovery sample (all *p* < 0.05), and three of them located at Chr4:47840038 (HR = 0.74, *p* = 0.015), Chr4:47839941 (HR = 0.80, *p* = 0.047), and Chr4:47839933 (HR = 0.82, *p* = 0.050) were also significantly associated with incident stroke in the replication sample. The gene-based association analysis found that DNA methylation of the 9 CpG sites at the *CORIN* promoter was jointly associated with stroke in both samples (all *p* < 0.05).

**Conclusion:**

DNA methylation levels of the *CORIN* gene promoter were lower in stroke patients and predicted a higher risk of incident stroke in Chinese adults. The underlying causality warranted further investigation.

## Introduction

Cardiac natriuretic peptides (NPs) including atrial natriuretic peptide (ANP) and B-type natriuretic peptide (BNP) play a critical role in the regulation of blood pressure and salt-water balance through natriuresis, diuresis, and vasodilatation (1). Their circulating levels have been associated with the risk of stroke which is the leading cause of long-term disability and mortality all over the world (2), China in particular (3), in various prospective studies (4–6). Corin, a trypsin-like protease highly expressed in the heart (7, 8), is the physiological activator of ANP (9) and could also activate BNP (10). It may be a switching regulator of the NP system and thereby contributing to the development of stroke. Indeed, the cardiovascular effect of corin has been suggested by basic and population studies. For example, the expression of corin was upregulated in atherosclerotic aorta intima and vascular endothelial cells stimulated by oxidative stress (11). Blood pressure was elevated in mice with *corin* gene knockout (12). In humans, single nucleotide variations (SNVs) in *CORIN*, the coding gene of corin protein, were associated with susceptibility to heart failure (13), cardiac hypertrophy (14), and hypertension (15). Circulating levels of corin have been associated with various cardiovascular disorders such as heart failure (16), atrial fibrillation (17), and myocardial infarction (18). Furthermore, our group previously found that decreased serum corin was significantly associated with prevalent stroke (19) and unfavorable poststroke outcomes (20). These findings suggest that corin could be a risk factor or drug candidate for the prevention and control of stroke. However, no inhibitor of corin function has been found in human plasma (21), which may increase the unsafety of its clinical translation. Therefore, a better understanding of the molecular mechanisms underlying the association between corin and stroke is urgent for clinical translation. As a mediator between the dynamic environment and fixed genome, DNA methylation may affect gene expression and function and thereby representing one of the candidate molecular mechanisms that we are seeking. To date, many DNA methylation markers of stroke have been identified by epigenome-wide association studies (22). DNA methylation levels of the global genome (23, 24) and some candidate genes, such as ATP-binding cassette G1 (25), matrix metalloproteinase-2 (26), estrogen receptor alpha (27), thrombomodulin (28), and tumor protein p53 (29) have been associated with stroke. As suggested above, we hypothesized that DNA methylation of the *CORIN* gene may also play a considerable role in stroke development, but lacking epidemiological evidence. Therefore, we aimed to examine the association between *CORIN* gene promoter methylation and the risk of stroke in two independent samples of Chinese adults. The identified methylation markers might be useful targets for the prevention and treatment of stroke because DNA methylation is a modifiable molecular modification.

## Methods

Methods of selection of study participants and data collection were described in the Supplementary material (eMethods) in detail. Following we briefly introduced the methods of the current study.

### Study participants

**Figure 1:**
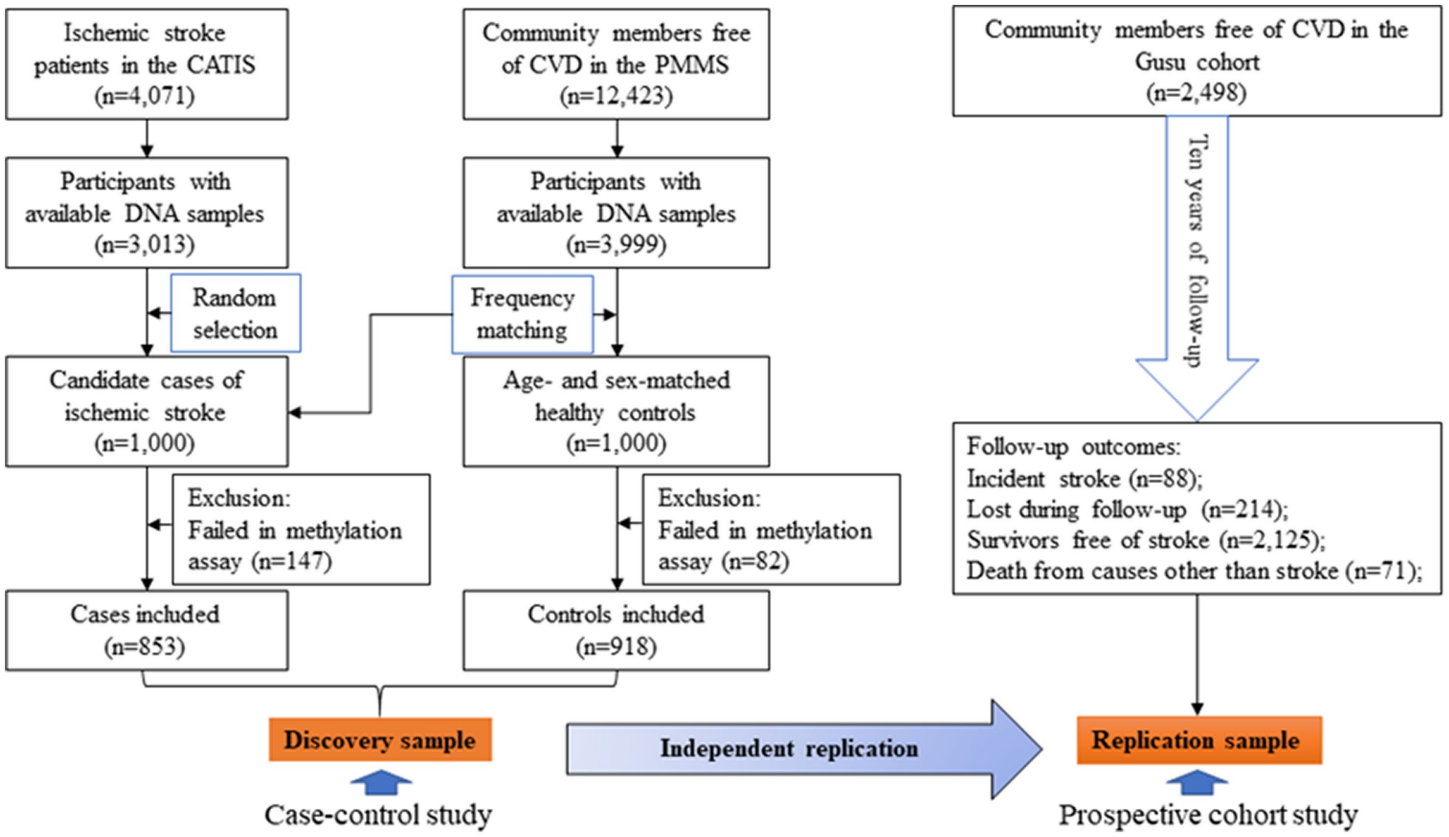
A flowchart illustrating the selection of study participants. CATIS, China Antihypertensive Trial in Acute Ischemic Stroke; CVD, cardiovascular disease; PMMS, Prevention of Metabolic syndrome and Multi-metabolic disorders Study.

The protocols of the present study were approved by the Soochow University Ethics Committee. The current study included 1,771 participants as the discovery sample and 2,498 participants as the replication sample. Figure 1 illustrates the selection of study participants. In brief, 1,000 patients were randomly selected as cases of ischemic stroke from the 3,013 patients with available DNA samples in the China Antihypertensive Trial in Acute Ischemic Stroke (CATIS) (30). By frequency matching, 1,000 age-and sex-matched controls were selected from the 3,999 community individuals free of cardiovascular diseases (CVD) and with available DNA samples in the Prevention of Metabolic syndrome and Multi-metabolic disorders Study (PMMS) (31). After excluding 229 (147 cases and 82 controls) participants whose samples failed in methylation quantification, 1,771 participants including 853 cases and 918 controls were finally included as the discovery sample. A total of 2,498 community members with 10 years of follow-up in the Gusu cohort were included as the replication sample. Written informed consent was obtained from all study participants.

### Quantification of *CORIN* promoter methylation

**Figure 2:**
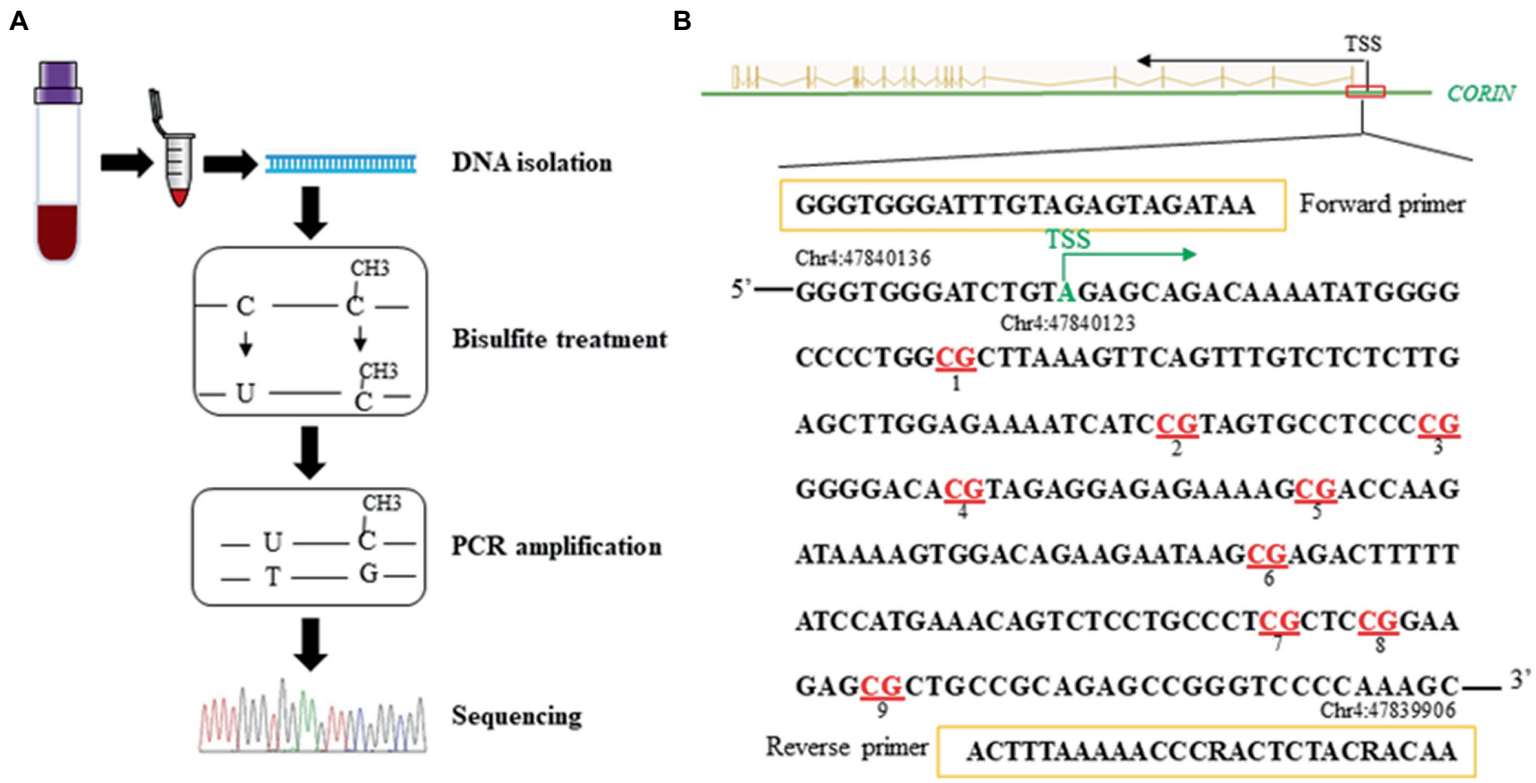
A schematic illustration of the methods **(A)** and the targeted sequence and primers **(B)** for targeted bisulfite sequencing. Red represents the CpG loci assayed in the *CORIN* gene promoter (+27 ~ +190 bp from TSS). TSS: transcriptional start site.

Genomic DNA was isolated from peripheral blood mononuclear cells in both samples. Levels of DNA methylation in the promoter region of the *CORIN* gene were quantified by targeted bisulfite sequencing (32). In brief, as illustrated in Figure 2, after bisulfite treatment, amplification by polymerase chain reaction (PCR), paired-end sequencing, and quality control, a total of 9 CpG loci in the *CORIN* promoter were assayed.

### Assessment of risk factors

In both samples, demographic data (age, sex, and education level), lifestyles (cigarette smoking and alcohol consumption), and metabolic factors (obesity, fasting glucose, blood lipids, and, blood pressure) were obtained by trained staff. Diabetes was defined as fasting glucose ≥7.0 mmol/L or self-reported history of diabetes (33). Hypertension was defined as SBP ≥140 mmHg and/or DBP ≥90 mmHg or under antihypertensive treatment in the last 2 weeks (34).

### Statistical analysis

The clinical characteristics of study participants were presented according to the status of stroke. Log2-transformation was applied to maximize the normality of data distribution for methylation levels at single CpG sites. The transformed data were used in downstream analyses. Both single CpG and joint associations between *CORIN* promoter methylation and stroke were repeatedly examined in both samples. All statistical analyses were performed using R Studio.

### Analysis of the discovery sample

The median levels of DNA methylation at single CpG sites were compared between patients with ischemic stroke and their healthy controls using the Wilcoxon rank-sum test. To examine the association between DNA methylation at a single CpG and stroke, we constructed a logistic regression model in which ischemic stroke (y/n) was the dependent variable and DNA methylation at each CpG site (after log2-transformation) was the independent variable, adjusting for potential confounding factors including age, sex, education level, hypertension, diabetes. Cigarette smoking, alcohol consumption, body mass index (BMI), low-density lipoprotein cholesterol (LDL-C), and high-density lipoprotein cholesterol (HDL-C). The false discovery rate (FDR) approach was applied to control multiple testing. To test the joint association between DNA methylation at multiple CpG sites and ischemic stroke, we first substituted the mean level of DNA methylation at multiple CpG sites for the methylation level of the targeted region and examined its association with ischemic stroke. Then, the weighted truncated product method (wTPM) was also applied by combining the raw *p*-values of single CpG associations, weighted on the regression coefficient (35).

### Analysis of the replication sample

To replicate and further examine whether *CORIN* promoter methylation at baseline predicted the risk of stroke incidence, we similarly examined the single CpG and gene-based associations between *CORIN* promoter methylation and stroke by constructing a competing-risks survival regression model. In this model, time (in years) to incident stroke was the dependent variable, baseline DNA methylation levels at each CpG site (after log2-transformation) was the independent variable, and death from causes other than stroke was the competing event, adjusting for the covariates listed above.

### Sensitivity analysis

To test whether the CpG sites identified can improve the predictive performance of the risk of stroke over traditional risk factors, we established and evaluated the predicting models fitted by the CpG methylation plus conventional risk factors versus conventional risk factors only. The net reclassification improvement (NRI) and integrated discrimination improvement (IDI) were calculated using the R packages “PredictABEL” and “nricens” in the discovery sample and “survIDINRI” in the replication sample.

## Results

### Clinical characteristics of participants

This study included 853 patients with ischemic stroke (mean aged 62 years, 53% men) and 918 healthy controls (mean aged 61 years, 55% men) as in the discovery sample and 2,498 participants (mean aged 53 years, 39% men) as the replication sample. Their clinical characteristics were presented in Table 1. In the discovery sample, cases of ischemic stroke as expected were more likely to be older and have more metabolic risk factors, such as hypertension, diabetes, lipids, and obesity than their healthy controls (all *p* < 0.05). In the replication sample, 88 participants developed stroke during follow-up. At baseline, they also had more risk factors listed above than those who remained free of stroke by the end of follow-up (all *p* < 0.05).

**Table 1:** Clinical characteristics of participants in the discovery and replication samples.

Characteristics	Discovery sample			Replication sample^*^		
	Healthy control	Ischemic stroke	*p*-value	Free of stroke	Incident stroke	*p*-value
No. of participants	918	853	–	2,410	88	
Age, years	61.2 ± 12.2	62.5 ± 12.1	0.026	52.4 ± 9.4	61.6 ± 8.9	<0.001
Sex, male (%)	503 (54.79)	453 (53.11)	0.507	926 (38.42)	36 (40.91)	0.719
Education level, high school or above (%)	580 (63.18)	732 (85.81)	<0.001	494 (20.50)	13 (14.77)	0.239
Current smoking, *n* (%)	348 (37.91)	294 (34.47)	0.145	558 (23.15)	24 (27.27)	0.442
Current drinking, *n* (%)	253 (27.56)	222 (26.03)	0.500	443 (18.38)	22 (25.00)	0.153
Hypertension, *n* (%)	325 (35.40)	670 (78.55)	<0.001	1,050 (43.57)	59 (67.05)	<0.001
Diabetes, *n* (%)	27 (2.94)	165 (19.34)	<0.001	203 (8.42)	14 (15.91)	0.024
Body mass index, kg/m^2^	22.36 ± 3.37	25.09 ± 3.39	<0.001	24.75 ± 3.59	25.67 ± 4.60	0.068
Systolic blood pressure, mmHg	134.6 ± 20.7	168.1 ± 16.8	<0.001	129.8 ± 17.0	138.2 ± 17.3	<0.001
Diastolic blood pressure, mmHg	80.0 ± 10.8	97.0 ± 10.7	<0.001	84.8 ± 9.3	86.2 ± 9.0	0.161
Fasting glucose, mmol/L	5.04 ± 1.18	6.78 ± 2.82	<0.001	5.38 ± 1.29	5.86 ± 2.32	0.057
Total cholesterol, mmol/L	4.66 ± 0.96	5.12 ± 1.16	<0.001	5.21 ± 1.77	5.38 ± 0.92	0.113
Triglycerides, mmol/L	1.56 ± 1.15	1.89 ± 4.96	0.054	1.46 ± 1.59	1.50 ± 1.40	0.798
LDL-cholesterol, mmol/L	3.01 ± 0.82	2.94 ± 0.98	0.115	2.99 ± 0.76	3.16 ± 0.77	0.048
HDL-cholesterol, mmol/L	1.34 ± 0.33	1.30 ± 0.41	0.032	1.51 ± 0.45	1.49 ± 0.38	0.686

All results were expressed with mean ± SD unless otherwise noted. LDL, low-density lipoprotein; HDL, high-density lipoprotein. ^*^The baseline characteristics of study participants in the replication sample were presented in participants who developed stroke or not during follow-up.

### Association between *CORIN* promoter methylation and stroke in the discovery sample

**Figure 3:**
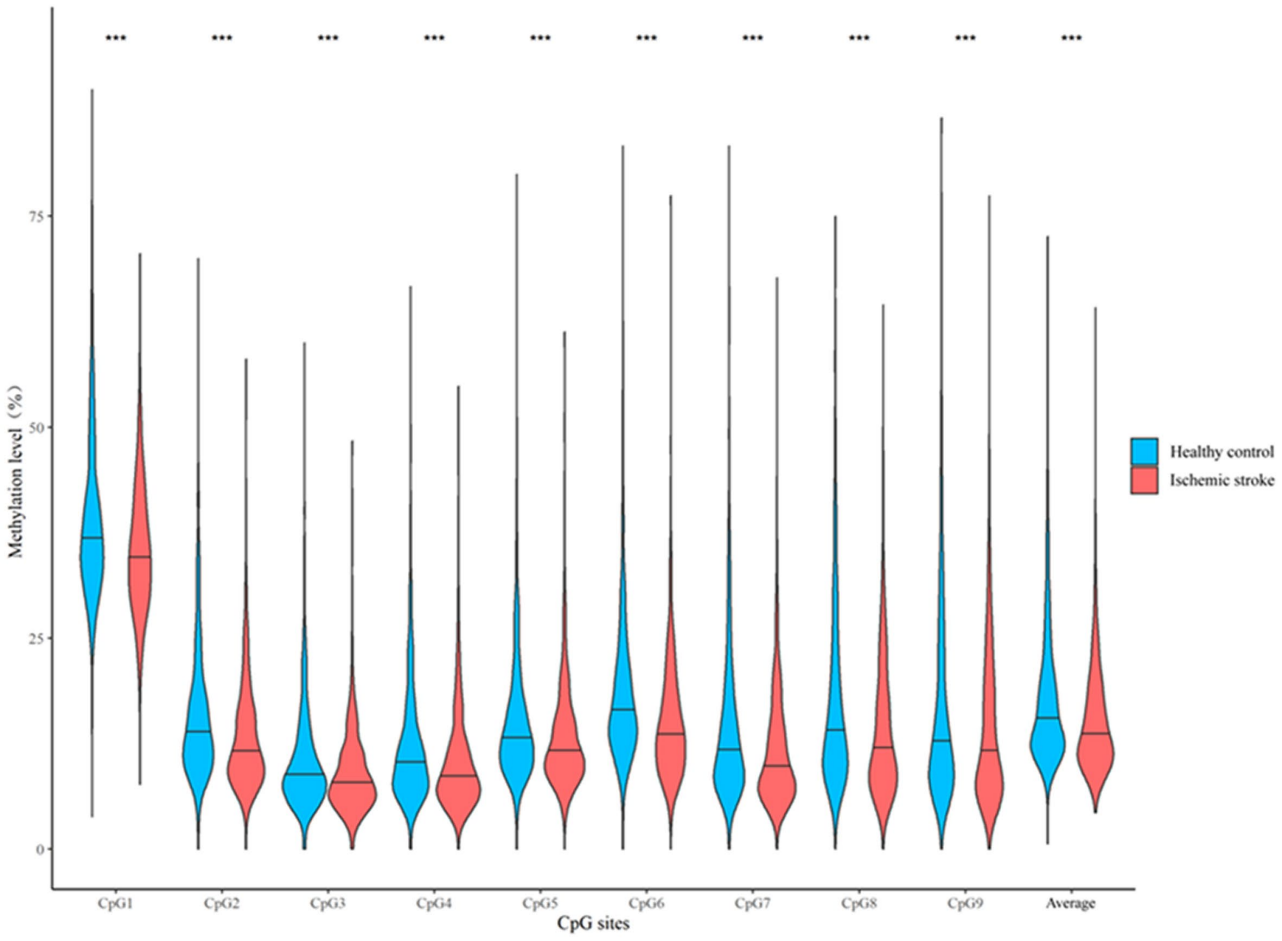
A box plot showing the individual and average DNA methylation levels of CpG sites in the *CORIN* promoter region according to the status of ischemic stroke in the discovery sample. ^***^p < 0.001.

As shown in Figure 3, DNA methylation levels at the 9 CpG sites assayed were all significantly lower in participants with ischemic stroke than in their healthy controls (all *p* < 0.05). After adjusting for confounding factors, DNA methylation levels at these CpG sites were also negatively associated with prevalent ischemic stroke (all *p* < 0.05, Table 2). These single CpG associations persisted after correction for multiple testing (all *q* < 0.05).

**Table 2:** The cross-sectional association between *CORIN* promoter methylation and ischemic stroke in the discovery sample.

CpG loci	Genomic position, GRCh37	Relative to TSS, bp	Methylation level, % Median (IQR)		OR (95%CI)^*^	*p*	q^†^
			Ischemic stroke	Healthy controls			
Single CpG association							
CpG1	Chr4:47840096	27	34.60 (30.13–40.16)	37.00 (32.55–42.86)	0.37 (0.26–0.52)	1.68E-08	3.35E-08
CpG2	Chr4:47840051	72	11.61 (8.77–16.31)	13.91 (10.45–18.90)	0.56 (0.46–0.67)	1.66E-09	7.45E-09
CpG3	Chr4:47840038	85	7.94 (5.86–11.12)	8.67 (6.52–12.82)	0.69 (0.58–0.81)	6.44E-06	7.89E-06
CpG4	Chr4:47840029	94	8.63 (6.37–12.50)	10.40 (7.36–14.98)	0.66 (0.56–0.78)	7.59E-07	1.14E-06
CpG5	Chr4:47840012	111	11.56 (9.15–15.76)	13.13 (10.27–17.78)	0.65 (0.53–0.78)	7.01E-06	7.89E-06
CpG6	Chr4:47839981	142	13.63 (10.24–18.52)	16.48 (12.99–22.13)	0.49 (0.40–0.60)	1.12E-12	1.01E-11
CpG7	Chr4:47839946	177	9.62 (6.95–15.00)	11.66 (8.27–18.06)	0.64 (0.55–0.75)	1.86E-08	3.35E-08
CpG8	Chr4:47839941	182	11.83 (8.11–18.67)	13.96 (9.71–22.42)	0.63 (0.54–0.73)	3.21E-09	9.62E-09
CpG9	Chr4:47839933	190	11.36 (7.36–19.81)	12.50 (8.43–22.77)	0.75 (0.65–0.86)	4.22E-05	4.22E-05
Gene-based association							
Average			13.61 (10.71–18.42)	15.48 (12.19–20.98)	0.50 (0.41–0.62)	3.38E-10	
wTPM						2.00E-04	

^*^Odds ratios indicate the risks of having ischemic stroke associated with a twofold increase in DNA methylation levels, adjusting for age, sex, education level, cigarette smoking, alcohol consumption, body mass index, low-and high-density lipoprotein cholesterol, hypertension, and diabetes. ^†^*q* values indicate the significance level after correction for multiple testing by the false discovery rate approach. GRCh37: Genome Reference Consortium Human Build 37; TSS, transcription start site; OR, odds ratio; CI, confidence interval; wTPM, weighted truncated product method; IQR, inter-quartile range.

We further examined whether hypermethylation at multiple CpG sites could be jointly associated with ischemic stroke. The mean methylation level of the 9 CpG sites was significantly lower in cases of ischemic stroke than in their healthy controls (median: 13.61% vs. 15.48%, *p* < 0.001, Figure 3). After multivariate adjustment for conventional risk factors, it was also significantly associated with a lower risk of prevalent ischemic stroke (OR = 0.50, *p* < 0.001 for log2-transformed methylation levels, Table 2). Similarly, the wTPM also found that DNA methylation of the 9 CpG sites in the *CORIN* promoter as a whole was significantly associated with ischemic stroke (*p* < 0.001).

### Association between *CORIN* promoter methylation and stroke in the replication sample

**Figure 4:**
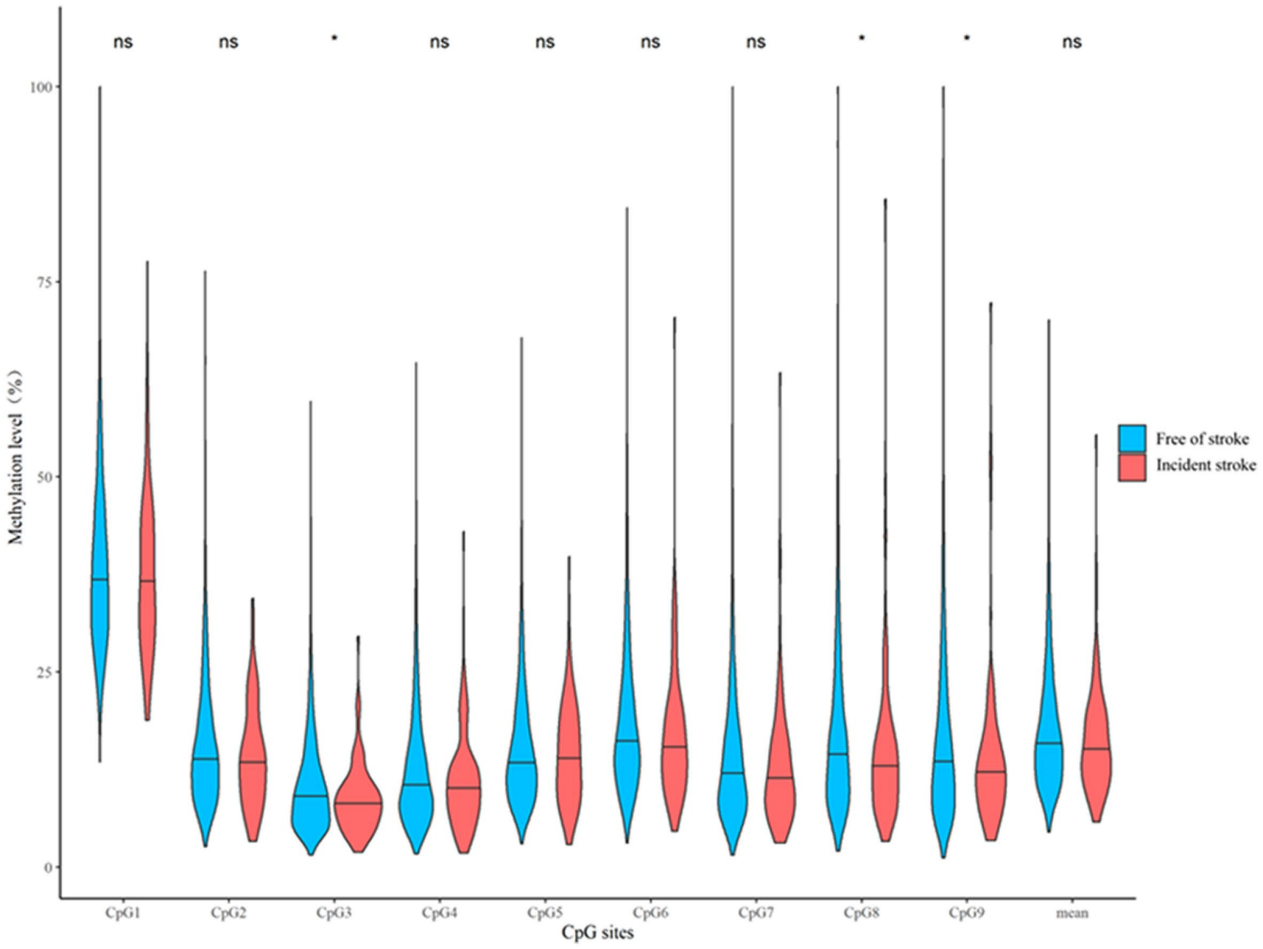
A box plot showing the individual and average DNA methylation levels of CpG sites in the *CORIN* promoter region according to the status of incident stroke in the replication sample. ^*^p < 0.05; ns: *p* > 0.05.

We further examined whether *CORIN* promoter methylation assayed in the discovery sample predicted the future risk of stroke in a prospective cohort study – the Gusu cohort as an independent replication sample. Participants who developed stroke during follow-up had lower median levels of DNA methylation at CpG3 (8.01% vs. 9.10%, *p* = 0.020), CpG8 (12.64% vs. 14.49%, *p* = 0.039), and CpG9 (12.03% vs. 13.50, *p* = 0.048) at baseline, compared to those who remained free of stroke by the end of follow-up (Figure 4). After adjusting for the same risk factors as the discovery sample, hypermethylation of these CpG sites seemed to be associated with a decreased risk of incident stroke during follow-up (Table 3). They were CpG3 located at Chr4:47840038 (HR = 0.74, *p* = 0.015), CpG8 located at Chr4:47839941 (HR = 0.80, *p* = 0.047), and CpG9 located at Chr4:47839933 (HR = 0.82, *p* = 0.050). Nevertheless, none of them survived multiple testing corrections (all *q* > 0.05).

**Table 3:** The prospective association between *CORIN* promoter methylation and stroke in the replication sample.

CpG loci	Genomic position, GRCh37	Relative to TSS, bp	Methylation level, % Median (IQR)		HR (95%CI)^*^	*p*	*q*^†^
			Developed stroke	Free of stroke			
Single CpG association							
CpG1	Chr4:47840096	27	35.98 (29.87–43.77)	36.88 (30.76–44.59)	0.86 (0.50–1.47)	5.77E-01	6.49E-01
CpG2	Chr4:47840051	72	13.54 (9.53–18.16)	13.83 (9.85–19.35)	0.80 (0.60–1.07)	1.33E-01	2.94E-01
CpG3	Chr4:47840038	85	8.01 (5.85–10.55)	9.10 (6.16–13.21)	0.74 (0.58–0.94)	1.53E-02	1.38E-01
CpG4	Chr4:47840029	94	10.10 (7.03–13.18)	10.54 (7.24–15.53)	0.86 (0.67–1.10)	2.28E-01	2.94E-01
CpG5	Chr4:47840012	111	14.05 (9.50–18.65)	13.39 (9.75–18.72)	0.95 (0.69–1.31)	7.45E-01	7.45E-01
CpG6	Chr4:47839981	142	15.16 (11.00–20.12)	16.24 (11.87–22.40)	0.83 (0.63–1.08)	1.68E-01	2.94E-01
CpG7	Chr4:47839946	177	10.76 (7.62–15.79)	12.06 (8.03–17.90)	0.87 (0.70–1.08)	2.09E-01	2.94E-01
CpG8	Chr4:47839941	182	12.64 (9.00–16.84)	14.49 (9.52–21.74)	0.80 (0.64–0.99)	4.65E-02	1.51E-01
CpG9	Chr4:47839933	190	12.03 (8.30–16.43)	13.50 (8.61–21.08)	0.82 (0.68–1.00)	5.04E-02	1.51E-01
Gene-based association							
Average			14.65 (11.42–19.14)	15.84 (11.98–21.37)	0.77 (0.56–1.06)	1.16E-01	
wTPM						2.67E-02	

^*^Risks of incident stroke associated with a twofold increase in DNA methylation levels at baseline, after adjusting for age, sex, education level, cigarette smoking, alcohol consumption, body mass index, and low-and high-density lipoprotein cholesterol, hypertension, and diabetes. ^†^*q* values indicate the significance level after correction for multiple testing by the false discovery rate approach. GRCh37, Genome Reference Consortium Human Build 37; TSS, transcription start site; HR, Hazard ratio; CI, confidence interval; wTPM, weighted truncated product method; IQR, inter-quartile range.

Although we did not find a significant association between the mean methylation level of the 9 CpG sites assayed and the risk of stroke (HR = 0.77, *p* = 0.116, Table 3), the wTPM found that DNA methylation at the 9 CpG sites was still jointly associated with the risk of future stroke in the replication sample (*p* = 0.027).

### Results of sensitivity analysis

The results from the discovery and replication samples consistently showed that DNA methylation levels at 3 CpG sites (CpG3, CpG8, CpG9) may be associated with stroke. Further, DNA methylation levels at these 3 CpG sites in the discovery sample, whereas only two of them (CpG3 and CpG9) in the replication sample could significantly improve the prediction performance over conventional risk factors as suggested by either NRI or IDI (Table 4).

**Table 4:** Discrimination for stroke risk prediction by *CORIN* promoter methylation over conventional risk factors.

Prediction models	Discovery sample			Replication sample		
	NRI	IDI	*p* for IDI	NRI	IDI	*p* for IDI
Conventional factors	reference	reference		reference	reference	
Conventional factors + CpG3	0.252 (0.160–0.343)	0.014 (0.008–0.019)	<0.001	0.172 (−0.031–0.446)	0.006 (0.001–0.017)	0.020
Conventional factors + CpG8	0.214 (0.123–0.306)	0.018 (0.011–0.024)	<0.001	0.204 (−0.055–0.415)	0.004 (0.000–0.013)	0.119
Conventional factors + CpG9	0.119 (0.027–0.211)	0.011 (0.006–0.016)	<0.001	0.313 (0.101–0.511)	0.004 (−0.001–0.014)	0.139

Conventional factors included age, sex, education level, cigarette smoking, alcohol consumption, body mass index, low- and high-density lipoprotein cholesterol, hypertension, and diabetes. NRI, net reclassification improvement; IDI, integrated discrimination index.

## Discussion

In Chinese adults participating in two independent samples, we examined for the first time the association between DNA methylation at the *CORIN* gene promoter and stroke. We found that hypermethylation at three CpG sites (located at Chr4:47840038, Chr4:47839941, and Chr4:47839933) was not only associated with lower odds of prevalent ischemic stroke but also predicted a lower future risk of stroke. DNA methylation levels at these CpG loci, the CpG located at Chr4:47840038 in particular, could increase the prediction power for the risk of stroke over conventional risk factors including behavioral and metabolic factors. Our results suggest that *CORIN* promoter methylation may play a potential role in the development of stroke through mechanisms beyond metabolic factors. Because DNA methylation is modifiable, *CORIN* promoter methylation may serve as a potential predictor or even probably a therapeutic target for stroke.

In line with our study, the potential role of corin in stroke has also been studied in previous studies. For example, a cell-based study found that the corin level was increased in cultured vascular endothelial cells stimulated by oxidized low-density lipoprotein, which is a major risk factor for atherosclerosis and stroke (11). In humans, *CORIN* gene polymorphisms have been associated with susceptibility to heart failure (13), cardiac hypertrophy (14), and hypertension (15). Clinical studies have found that circulating levels of corin were associated with heart failure (16), atrial fibrillation (17), and myocardial infarction (18), as well as major adverse cardiovascular events after the onset of heart failure (36) and acute myocardial infarction (37), all of which shared many pathological mechanisms with stroke. Our previous case–control study including 597 stroke patients and 2,498 community-based healthy controls provided the first evidence for the association between serum corin levels and stroke (19). Recently, we further revealed a significant prospective association between serum corin at baseline and 10-year risk of stroke in the Gusu cohort (38). These findings consistently suggested a potential role of corin protein in stroke, but the molecular mechanisms through which corin contributes to stroke are not very clear.

DNA methylation is a modifiable chemical modification of the genome without changing the gene sequence and could repress gene expression by recruiting proteins associated with gene suppression or by preventing transcription factors from binding to DNA (39). Indeed, many risk factors of stroke have been associated with the level of DNA methylation, such as smoking (40), obesity (41), and hypertension (42). Compared with questionnaires, DNA methylation may better capture the influence of environmental factors and individual behavioral habits (which are difficult to quantify accurately) on the risk of stroke, therefore, DNA methylation may provide a more accurate prediction of stroke risk (43). It has been suggested to be involved in the pathogenesis of stroke in some small case–control studies. For example, epigenome-wide association studies (EWAS) have identified multiple epigenetic markers for stroke (44–46). Clinical studies found that DNA promoter methylation at some candidate genes, such as tumor necrosis factor (47), estrogen receptor alpha (27), and matrix metalloproteinase-2 (26) were associated with stroke. In a study including a discovery sample of 511 patients with first-ever acute ischemic stroke and a replication sample of 85 patients with the same disease, biological age has been estimated based on DNA methylation at 71 CpG sites, and was found to be an independent predictor of 3-month ischemic stroke outcome evaluated by 3-month modified Rankin Scale (48), another study reported that biological age calculated by DNA methylation was associated with death within 3 months after ischemic stroke (49). Therefore, DNA methylation of the *CORIN* gene may exist as a potential molecular mechanism that regulates the expression or excretion of corin protein and thereby participates in the pathogenesis of stroke. Indeed, a case–control study including 731 hypertension patients and 731 controls has demonstrated that *CORIN* gene methylation mediated the effect of *CORIN* SNVs on corin protein level (31). To the best of our knowledge, no study examined the association between DNA methylation at the *CORIN* gene and the risk of stroke. Leveraging two independent samples, our study provided initial evidence that *CORIN* promoter methylation may play an important role in the development of stroke.

Previous studies introduced that the contribution of methylation at single CpG sites to a complex phenotype was relatively small (50, 51). This phenomenon was also observed in our study. DNA methylation levels of individual CpG sites at baseline could only explain 0.04–2.46% of the risk of stroke during follow-up. Although DNA methylation levels at the 9 CpG sites assayed were strongly correlated, only three CpG sites were associated with stroke, and none of them survived correction for multiple testing. Although a small effect size is not easy to be identified, the joint effect of multiple CpG sites may be stronger and more suitable for stroke risk prediction. Therefore, we examined the joint association between methylation of multiple CpG sites in the *CORIN* promoter with incident stroke and found a significant joint association. Our results indicated the importance of detecting the combined effect of methylation at multiple CpG sites on complex disorders.

The strengths of our study include independent replication, comprehensive measurement and adjustment for confounding factors, and application of weighted truncated product methods to test the joint association between *CORIN* promoter methylation and stroke. Some limitations need to be acknowledged. First, as an observational study, unknown confounders may influence the association that we found. The causality between *CORIN* promoter methylation and stroke is still unclear. Second, our participants only included Chinese adults. The generalizability of our results to other populations with different ethnic backgrounds is uncertain. Third, the methylation we detected came from peripheral blood genomic DNA. It is not clear whether it can reflect the effect of methylation in the target organs of stroke, such as the brain and arteries. However, there is increasing evidence that epimutations may not be limited to affected tissues, but can also be detected in peripheral blood (52). Fourth, whether *CORIN* promoter methylation affects gene expression and protein synthesis is still unknown and needs further investigation. Fifth, natriuretic peptide, the final activation hormone of the natriuretic peptide system to exert cardiovascular protection, may be affected by the methylation level of the *CORIN* gene, however, we did not have data on natriuretic peptides and could not include the levels of natriuretic peptide in this study.

## Conclusion

In summary, hypermethylation of the *CORIN* gene promoter was not only associated with prevalent ischemic stroke but also predicted a lower future risk of incident stroke in Chinese adults. DNA methylation level of the *CORIN* promoter, the CpG located at Chr4:47840038 in particular, could be a predictor of incident stroke. Because DNA methylation is a modifiable molecular modification, *CORIN* promoter methylation could be a candidate therapeutic target for the prevention and management of stroke, although the underlying causality is still unclear.

## Data availability statement

The original contributions presented in the study are publicly available. This data can be found at: https://doi.org/10.5061/dryad.k3j9kd5bx.

## Ethics statement

The studies involving human participants were reviewed and approved by the Soochow University Ethics Committee. The patients/participants provided their written informed consent to participate in this study.

## Author contributions

LC and JJ performed the data analysis and drafted the manuscript. HP and QZ developed the concept of the study design and revised the manuscript. JY, YL, and XZ obtained the clinical data and critically reviewed the manuscript. JJ, QZ, and HP contributed to the interpretation of the results. All authors read and approved the final manuscript.

## Funding

This study was supported by the National Natural Science Foundation of China (nos. 82173596, 81903384, and 81872690), the Youth Program of Science and Technology for Invigorating Health through Science and Education in Suzhou (no. GSWS2019091), Suzhou Key Technologies of Prevention and Control of Major Diseases and Infectious Diseases (no. GWZX202001), and a Project of the Priority Academic Program Development of Jiangsu Higher Education Institutions.

## Conflict of interest

The authors declare that the research was conducted in the absence of any commercial or financial relationships that could be construed as a potential conflict of interest.

## Publisher’s note

All claims expressed in this article are solely those of the authors and do not necessarily represent those of their affiliated organizations, or those of the publisher, the editors and the reviewers. Any product that may be evaluated in this article, or claim that may be made by its manufacturer, is not guaranteed or endorsed by the publisher.
