# Supplementary material for: Association between CORIN promoter methylation and stroke: Results from two independent samples of Chinese adults
Source: Front Neurol. 2023 Mar 31;14:1103374. doi: 10.3389/fneur.2023.1103374 (PMC10102360; doi:10.3389/fneur.2023.1103374)
Supplement: Supplementary file 1 [file Data_Sheet_1.DOCX]

**Supplementary data**

**eMETHODS**

**Study participants**

The current study included 1,771 participants as the discovery sample and 2,498 participants as the replication sample. **Figure 1** illustrates the selection of study participants.

**Discovery sample:** The China Antihypertensive Trial in Acute Ischemic Stroke (CATIS) was a multicenter randomized clinical trial (clinicaltrials.gov Identifier: NCT01840072) designed to test whether blood pressure reduction within the first 48 hours after the onset of an acute ischemic stroke would reduce death and major disability at 14 days or hospital discharge ^1^. The study protocols were approved by the institutional review boards at Soochow University in China and Tulane University in the United States, as well as the ethical committees of all participating hospitals. After providing written informed consent, 4,071 patients aged over 22 years with first-ever IS were recruited. Among them, 3,013 patients provided their blood samples and agreed to participate in the subsequent genetic studies with voluntary principles. From the 3,013 patients with available DNA samples, 1000 patients were randomly selected as cases of IS in the present study. By frequency matching, 1,000 age- and sex-matched controls were selected from the 3,999 community individuals free of cardiovascular diseases (CVD) and with available DNA samples participating in the Prevention of Metabolic syndrome and Multi-metabolic disorders Study (PMMS) ^2^, a community-based prospective cohort study of CVD and its risk factors in Chinese adults. The protocols of the PMMS were approved by the Soochow University Ethics Committee. Written informed consent was obtained from all study participants. After excluding 229 (147 cases and 82 controls) participants whose samples failed in methylation quantification, a total of 1,771 participants including 853 IS cases and 918 healthy controls were finally included as the discovery sample.

**Replication sample:** The Gusu cohort is a community-based prospective longitudinal study of CVD and its risk factors in middle-aged and elderly Chinese adults. The study design, survey methods, and laboratory techniques have been described previously ^3^. In brief, a total of 2,498 community members over 30 years free of overt CVD completed the baseline examination in 2010. During 10 years of follow-up through 2020, 88 participants developed stroke, 71 participants died from causes other than stroke, and 214 participants were lost. Follow-up outcomes were confirmed by an endpoint review committee. The date of each event was ascertained from either the initial point of diagnosis or a death certificate. The protocols of the Gusu cohort were approved by the Soochow University Ethics Committee. Written informed consent was obtained from all study participants.

**Quantification of *CORIN* promoter methylation**

As illustrated in **Figure** 2, DNA methylation levels in the promoter region of the *CORIN* gene were quantified by targeted bisulfite sequencing as previously described ^4^, using genomic DNA isolated from peripheral blood mononuclear cells in both samples. In brief, based on the genomic coordinates of the *CORIN* promoter in Genome Reference Consortium Human Build 37 (GRCh37), we carefully designed the primers to detect the maximum CpG loci within the CpG islands. The region of Chr4: 47840136 – 47839906 (relative to TSS: -27 bp to +190 bp) was finally targeted. Following primer validation, genomic DNA was bisulfite-treated using the EZ DNA Methylation-Gold Kit (Zymo Research, Inc., CA, United States) according to the manufacturer’s protocol, which converts unmethylated cytosine into uracil, while methylated cytosine does not change. The treated samples were amplified, barcoded, and sequenced by Illumina Hiseq 2000 (Illumina, Inc., CA, United States) using the paired-end sequencing protocol according to the manufacturer’s guidelines. The methylation level at each CpG dinucleotide was calculated as the percentage of the methylated alleles over the sum of methylated and unmethylated alleles. For quality control, the samples with bisulfite conversion rate <98% and the cytosine sites with an average coverage of less than 20× were filtered out. DNA methylation levels were finally quantified at 9 CpG loci in the *CORIN* promoter.

**Assessment of risk factors**

In both samples, demographic data including age, sex, and education level, and lifestyles including cigarette smoking and alcohol consumption were obtained by questionnaires administered by trained staff. Cigarette smoking was defined as current smoking or not. Current smokers were defined as those who have smoked at least 100 cigarettes in their entire life, have smoked cigarettes regularly, and smoking currently. Alcohol consumption was classified as current drinkers or not. Current drinkers were those who had consumed any alcoholic beverage ≥12 times during the past year. Body weight (kg) and height (cm) were measured when participants wore light clothes and no shoes by trained staff. Body mass index (BMI) was calculated by dividing weight in kilograms by the square of height in meters (kg/m^2^). Fasting glucose and blood lipids including total cholesterol, triglycerides, high-density lipoprotein cholesterol (HDL-C), and low-density lipoprotein cholesterol (LDL-C), were measured by standard laboratory methods ^3^. Diabetes was defined as fasting glucose ≥7.0 mmol/L or self-reported history of diabetes ^5^. Three blood pressure measurements were performed by trained staff using a standard mercury sphygmomanometer and a cuff of appropriate size, according to a common protocol ^6^, after the participants had been resting for at least 5 min in a relaxed, sitting position. The first and fifth Korotkoff sounds were recorded as systolic blood pressure (SBP) and diastolic blood pressure (DBP), respectively. The means of the three measurements were used for statistical analyses. Participants with an SBP ≥140 mmHg and/or a DBP ≥90 mmHg or under antihypertensive treatment in the last 2 weeks were diagnosed with hypertension, according to the Chinese guidelines for the management of hypertension ^7^.

**Statistical analysis**

The clinical characteristics of study participants were presented according to the status of stroke. Log2-transformation was applied to maximize the normality of data distribution for methylation levels at single CpG sites. The transformed data were used in downstream analyses. Both single CpG and gene-based associations between *CORIN* promoter methylation and stroke were repeatedly examined in both samples. All statistical analyses were performed using R Studio.

**Analysis of the discovery sample**

The median levels of DNA methylation at single CpG sites were compared between patients with ischemic stroke and their healthy controls using the Wilcoxon rank-sum test. To examine the single CpG association between *CORIN* promoter methylation and stroke, we constructed a logistic regression model in which ischemic stroke (y/n) was the dependent variable and DNA methylation at each CpG site (after log2-transformation) was the independent variable, adjusting for age, sex, education level, cigarette smoking, alcohol consumption, BMI, LDL-C, HDL-C, hypertension, and diabetes. Multiple testing was controlled by adjusting for the total number of CpG loci tested using the false discovery rate (FDR) approach, and an FDR-adjusted *P*-value (i.e., *q* value) of less than 0.05 was considered statistically significant. To test the joint association between DNA methylation at multiple CpG sites in the *CORIN* promoter region and ischemic stroke, we substituted the average methylation level at multiple CpG sites for the methylation level at the targeted region and examined its association with ischemic stroke. The weighted truncated product method (wTPM), which combines the raw *P*-values of all CpG sites with a preselected threshold (e.g., raw *P* < 0.1 in this study) was also applied. The regression coefficient of each CpG methylation was included as the weight in the wTPM ^8^. This method has been evaluated by simulation studies ^9^ and applied to epigenetic analysis ^10^.

**Analysis of the replication sample**

To replicate and further examine whether *CORIN* promoter methylation at baseline predicted the risk of stroke incidence, we similarly examined the single CpG and gene-based associations between *CORIN* promoter methylation and stroke by constructing a competing-risks survival regression model. In this model, time (in years) to incident stroke was the dependent variable, baseline DNA methylation levels at each CpG site (after log2-transformation) was the independent variable, and death from causes other than stroke was the competing event, adjusting for the covariates listed above. The competing-risks survival regression models were constructed by the R package “cmprsk”.

**Sensitivity analysis**

To test whether the CpG sites identified can improve the predictive performance of the risk of stroke over traditional risk factors including age, sex, education level, cigarette smoking, alcohol consumption, BMI, LDL-C, HDL-C, hypertension, and diabetes, we established and evaluated the predicting models fitted by the CpG methylation plus conventional risk factors versus conventional risk factors only. The net reclassification improvement (NRI) and integrated discrimination improvement (IDI) were calculated using the R packages “PredictABEL” and "nricens" in the discovery sample and "survIDINRI" in the replication sample.

**eReference:**

1. He J, Zhang Y, Xu T, Zhao Q, Wang D, Chen CS, et al. Effects of immediate blood pressure reduction on death and major disability in patients with acute ischemic stroke: The catis randomized clinical trial. *JAMA*. 2014;311:479-489

2. Zhang H, Mo X, Qian Q, Zhou Z, Zhu Z, HuangFu X, et al. Associations between potentially functional corin snps and serum corin levels in the chinese han population. *BMC Genetics*. 2019;20

3. Peng H, Zhang Q, Cai X, Liu Y, Ding J, Tian H, et al. Association between high serum soluble corin and hypertension: A cross-sectional study in a general population of china. *Am J Hypertens*. 2015;28:1141-1149

4. Pu W, Wang C, Chen S, Zhao D, Zhou Y, Ma Y, et al. Targeted bisulfite sequencing identified a panel of DNA methylation-based biomarkers for esophageal squamous cell carcinoma (escc). *Clin Epigenetics*. 2017;9:129

5. American Diabetes A. 2. Classification and diagnosis of diabetes: Standards of medical care in diabetes-2019. *Diabetes Care*. 2019;42:S13-S28

6. Chobanian AV, Bakris GL, Black HR, Cushman WC, Green LA, Izzo JL, et al. The seventh report of the joint national committee on prevention, detection, evaluation, and treatment of high blood pressure: The jnc 7 report. *JAMA*. 2003;289:2560-2572

7. Liu L-S. [2010 chinese guidelines for the management of hypertension]. *Zhonghua Xin Xue Guan Bing Za Zhi*. 2011;39:579-615

8. Zaykin DV, Zhivotovsky LA, Westfall PH, Weir BS. Truncated product method for combining p-values. *Genet Epidemiol*. 2002;22:170-185

9. Sheng X, Yang J. Truncated product methods for panel unit root tests. *Oxf Bull Econ Stat*. 2013;75:624-636

10. Peng H, Zhu Y, Strachan E, Fowler E, Bacus T, Roy-Byrne P, et al. Childhood trauma, DNA methylation of stress-related genes, and depression: Findings from two monozygotic twin studies. *Psychosom Med*. 2018;80:599-608
